# CliniCAM: A Technical Report of a Mobile Health Application for Structured Clinical Image Documentation and Tag-Based Dataset Generation

**DOI:** 10.7759/cureus.108942

**Published:** 2026-05-15

**Authors:** Addiel U De Alba Solis, Eduardo Gómez Sánchez

**Affiliations:** 1 General Medicine, University of Guadalajara, Guadalajara, MEX; 2 Clinical Medicine, University of Guadalajara, Guadalajara, MEX

**Keywords:** clinical data management, clinical data science, clinical decision support, clinical image, cloud infrastructure, dataset, digital health technology, image in healthcare, mobile apps (mhealth), photo-documentation

## Abstract

Clinical image recording is often poorly standardized. The use of personal devices and messaging platforms results in data fragmentation and limited accessibility. Previous mobile solutions prioritized secure image capture and electronic health record integration, yet offered minimal support for structured organization and efficient retrieval. This report describes the design and development of CliniCAM, a mobile health application for structured clinical image capture, annotation, tagging, retrieval, and dataset generation. CliniCAM was developed using FlutterFlow (FlutterFlow Inc., Mountain View, CA, USA) with a Firebase backend (Google, Mountain View, CA, USA). Firestore manages structured data, and Firebase Storage handles image management. The application supports in-app image capture, patient association, free-text annotation, and assignment of custom tags. Users can search by patient, free-text, or tag. Data export is enabled through Google Sign-In and the Google Drive API, allowing the generation of datasets containing images and metadata in JSON format.

CliniCAM delivers a unified workflow for clinical image documentation by integrating image capture, metadata annotation, and tag-based classification within a mobile interface. The system permits efficient retrieval and supports the creation of tagged datasets for secondary applications. For example, in an orthopedic consultation, a clinician can use CliniCAM to capture high-resolution images of musculoskeletal findings, such as joint deformities, surgical wounds, or traumatic injuries, directly within the app, tag the images with terms such as “osteoarthritis” or “fracture,” and enter relevant clinical annotations. These images are immediately associated with the patient record and securely stored. During follow-up visits, the clinician can quickly retrieve previous images using patient identifiers or tags to monitor disease progression. Similarly, in wound care, nurses can document wound healing over time, with images organized by anatomical site and wound type, thereby facilitating clinical decision-making and generating datasets for quality improvement or research. CliniCAM delivers a scalable and affordable solution for structured clinical image documentation. The tag-based system enables dataset generation at the point of care, addressing limitations within traditional storage systems and supporting future research, educational efforts, and artificial intelligence applications.

## Introduction

Clinical photography is essential in modern medicine, especially in specialties such as dermatology, wound care, and orthopedics [1]. Despite the widespread availability of high-resolution smartphone cameras, clinical image documentation remains fragmented and inconsistently structured [2]. In numerous clinical settings, images are captured on personal devices and stored in non-standardized formats, which restricts accessibility, traceability, and clinical utility [3]. While existing mobile solutions address certain limitations through secure capture and electronic health record (EHR) integration, these systems mainly serve as storage and archival tools, offering limited support for organization and retrieval [3]. Additionally, many existing workflows offer limited support for longitudinal, side-by-side image comparison during follow-up care, thereby reducing their utility for monitoring disease progression over time.

There is a continued need for tools that facilitate secure image capture and enable clinicians to efficiently organize, retrieve, and reuse clinical images. Lightweight and flexible systems are particularly necessary in academic and resource-limited environments [4].

CliniCAM was developed to tackle these challenges by providing a mobile system that integrates image capture, annotation, tag-based classification, and dataset generation within a unified workflow.

## Technical report

System architecture and technical design

CliniCAM is a cross-platform mobile application developed with FlutterFlow (FlutterFlow Inc., Mountain View, CA, USA), supporting rapid development and ensuring a consistent user interface across devices. Backend services include Firebase (Google, Mountain View, CA, USA), with Firestore managing structured data and Firebase Storage handling images.

**Figure 1:**
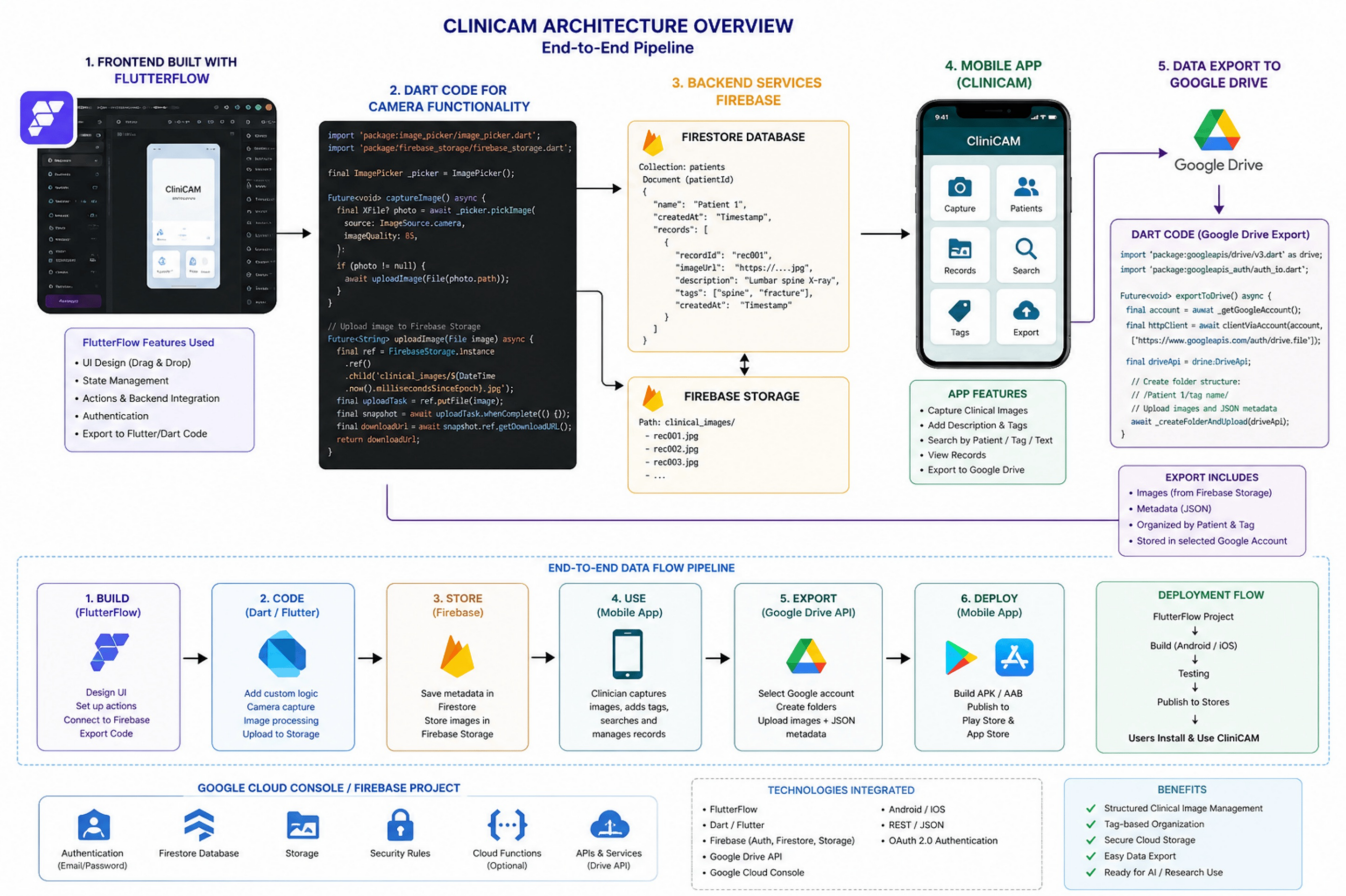
End-to-end system architecture of CliniCAM illustrating mobile application development with FlutterFlow, Dart-based camera integration, Firebase backend services (Firestore and Storage), and dataset export to Google Drive Image Credit: Authors using Canva (Canva Pty Ltd., Sydney, Australia)

The system operates in a cloud-based environment. Clinical images and metadata are captured at the point of care and synchronized with cloud services in real time, supporting scalability, centralized management, and multi-device access. The modular cloud-based architecture may also support future integration with institutional EHR systems, such as Epic and Cerner, through interoperability frameworks, including HL7 FHIR. The end-to-end architecture of CliniCAM, including FlutterFlow development, Firebase integration, and Google Drive export, is illustrated in Figure 1.

Data model and structure

Clinical records are stored as structured objects containing patient identifiers, secure image references, free-text clinical descriptions, user-defined tags, and timestamps associated with image acquisition. User-defined tags are dynamically created and stored as arrays to enable flexible classification and retrieval of clinical cases. The absence of predefined schemas allows adaptation across different specialties and workflows while supporting scalable dataset generation.

Image acquisition and storage

Images are captured directly within the application using an integrated camera interface, reducing reliance on device-native galleries.

**Figure 2:**
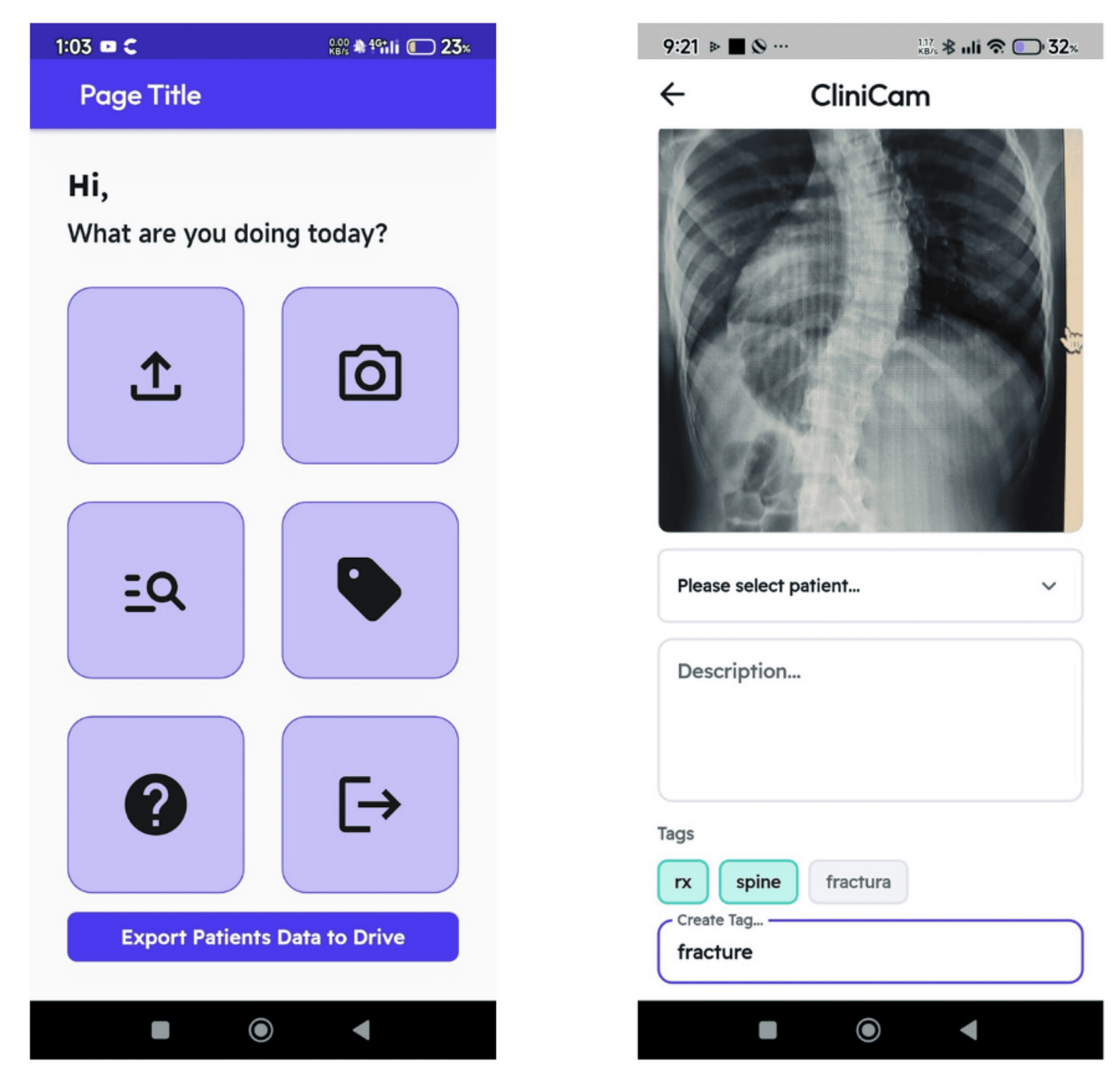
Main interface of the CliniCAM mobile application displaying core functionalities, including image capture, data upload, search, tag management, and dataset export

Captured images are uploaded to Firebase Storage, and each image is linked to its corresponding metadata in Firestore via a secure URL. Separating media from metadata enables efficient queries and prevents data duplication. This structure also facilitates longitudinal follow-up comparisons by enabling sequential image retrieval for individual patients and diagnostic tags. The application's primary user interface and core functionalities are shown in Figure 2.

Search and retrieval system

CliniCAM uses a query-based retrieval system in Firestore, allowing users to search records through patient identifiers, free-text clinical descriptions, and diagnostic tags. Tag-based retrieval is a core feature of the platform, enabling rapid access to clinically relevant cases and transforming stored images into searchable, structured datasets.

Authentication and security considerations

User access is managed through credential-based authentication within the application. Google authentication is additionally utilized for data export.

Data storage and transmission adhere to Firebase security policies, including encryption and access controls. Although CliniCAM is currently a prototype system, these features provide a foundational framework for secure clinical data management. Future development will focus on aligning with healthcare regulatory standards, including the Health Insurance Portability and Accountability Act (HIPAA) and the General Data Protection Regulation (GDPR) requirements, to support broader clinical implementation. Future development will further explore institutional authentication workflows and role-based access management to enhance enterprise-level security and interoperability.

Data export and interoperability

**Figure 3:**
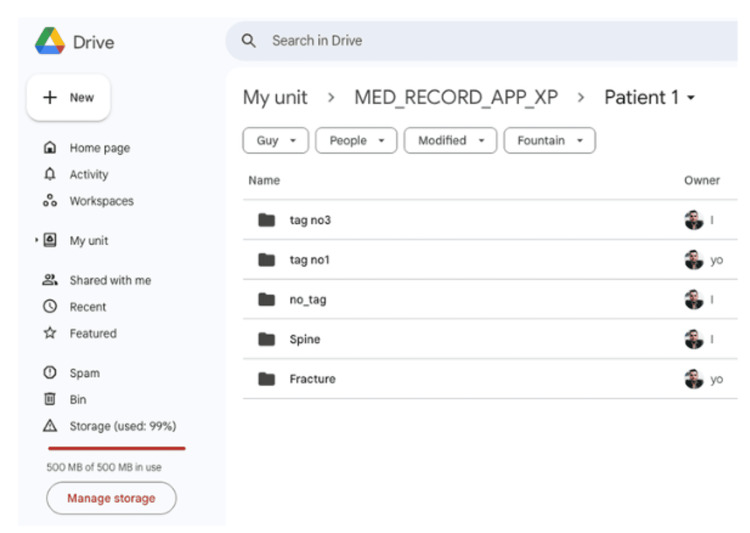
Hierarchical folder structure of exported clinical datasets in Google Drive, demonstrating tag-based classification and patient-level organization

Exported datasets contain clinical images alongside associated metadata, including descriptions, tags, and timestamps. Data are hierarchically organized according to patient and diagnostic category before export to Google Drive (Google, Mountain View, CA, USA). Images and metadata are exported in JSON-compatible structures to support interoperability with research workflows, educational repositories, and future machine-learning applications. The exported structured datasets may additionally support longitudinal outcome tracking, educational repositories, and future validation studies involving artificial intelligence workflows. The hierarchical organization of exported datasets within Google Drive is presented in Figure 3.

Design rationale

The system favors accessibility, scalability, and ease of use. FlutterFlow supports rapid, iterative development, while Firebase provides integration, real-time synchronization, and expandability.

A flexible tagging system replaces preset schemas, supporting adaptability across specialties and enabling dataset generation during routine workflows. To enhance interoperability and facilitate future research applications, we plan to align user-defined tags with established medical ontologies such as the International Classification of Diseases. This approach will allow tags created within CliniCAM to be mapped to standard terminologies, promoting consistent information exchange and integration with other clinical systems. Ongoing development will explore mechanisms for ontology integration and optional tag standardization within the app. Future development may also incorporate standardized terminology mapping through SNOMED CT and HL7 FHIR-compatible metadata structures to facilitate broader interoperability across healthcare systems.

## Discussion

This report presents the development of CliniCAM, a mobile-based platform for structured clinical image documentation and dataset generation. Previous mobile systems for clinical photography have primarily focused on secure image acquisition, authentication, and integration with electronic health record infrastructures [2,3]. Although these approaches improve centralization and compliance, many still rely on static storage models that offer limited support for dynamic organization and image retrieval.

CliniCAM addresses these limitations by incorporating tag-based classification, structured metadata generation, and query-based retrieval. By enabling clinicians to organize images using flexible diagnostic tags and searchable annotations, the platform transforms clinical image storage from passive archival into an actively structured dataset. This capability is particularly relevant in academic and resource-constrained environments where lightweight, modular systems may be preferable to enterprise-scale infrastructure [4].

The export architecture of CliniCAM further distinguishes the system from traditional mobile image-capture applications. By integrating with the Google Drive API, the platform enables exporting images and metadata in JSON-compatible formats, organized by patient and diagnostic category. This functionality facilitates secondary applications, including educational repositories, longitudinal case tracking, quality improvement initiatives, and future artificial intelligence development. Although the current report focuses primarily on technical implementation and workflow architecture, future studies should evaluate usability, workflow efficiency, user satisfaction, and comparative performance in real-world clinical environments. The increasing relevance of structured annotation systems and interoperable biomedical datasets has been highlighted in recent medical informatics literature [5,6].

Additionally, the use of FlutterFlow and Firebase enabled rapid prototyping and scalable cloud deployment while maintaining interoperability and multi-device synchronization. The modular architecture may support future integration with standardized healthcare interoperability frameworks, such as HL7 FHIR, and ontology-based tagging systems, including SNOMED CT and ICD terminology. Prospective pilot studies and implementation analyses will be important to assess scalability, clinician adoption, and integration into routine clinical workflows across multiple specialties.

## Conclusions

CliniCAM is a mobile application for structured clinical image documentation that integrates tagging and dataset generation. This system enables clinicians to create organized, searchable datasets and addresses major limitations of previous approaches. The platform may support applications in clinical practice, education, and data-driven tools such as artificial intelligence. The platform’s flexible architecture may additionally support future interoperability with enterprise clinical systems and standardized biomedical data frameworks. Although initial user experiences with secure mobile apps for clinical image capture have been documented, additional research is needed to evaluate their effectiveness in real-world clinical settings.
